# Poor patient outcome correlates with active engulfment of cytokeratin positive CTCs within cancer-associated monocyte population in lung cancer

**DOI:** 10.1007/s10585-024-10270-w

**Published:** 2024-02-28

**Authors:** A. P. Wiegmans, E. Ivanova, V. Y. Naei, J. Monkman, J. Fletcher, W. Mullally, M. E. Warkiani, K. O’Byrne, A. Kulasinghe

**Affiliations:** 1 Cancer and Ageing Research Program, Centre for Genomics and Personalised Health, Queensland University of Technology, Woolloongabba, QLD 4102 Australia; 2 Princess Alexandra Hospital, Oncology, Woolloongabba, QLD 4102 Australia; 3 School of Biomedical Engineering, University of Technology Sydney, Sydney, Australia; 4 Faculty of Medicine, Frazer Institute, The University of Queensland, Brisbane, QLD Australia

**Keywords:** Lung cancer, Cancer-associated macrophage-like cells (CAMLs), Tumour associated monocytes (TAM), Cytokeratins, Circulating tumour cells (CTCs), Leucocyte common antigen

## Abstract

High rates of mortality in non-small cell lung cancer lung cancer is due to inherent and acquired resistance to systemic therapies and subsequent metastatic burden. Metastasis is supported by suppression of the immune system at secondary organs and within the circulation. Modulation of the immune system is now being exploited as a therapeutic target with immune checkpoint inhibitors. The tracking of therapeutic efficacy in a real-time can be achieved with liquid biopsy, and evaluation of circulating tumour cells and the associated immune cells. A stable liquid biopsy biomarker for non-small cell lung cancer lung cancer has yet to be approved for clinical use. We performed a cross-sectional single-site study, and collected liquid biopsies from patients diagnosed with early, locally advanced, or metastatic lung cancer, undergoing surgery, or systemic therapy (chemotherapy/checkpoint inhibitors). Evaluation of overall circulating tumour cell counts, or cluster counts did not correlate with patient outcome. Interestingly, the numbers of Pan cytokeratin positive circulating tumour cells engulfed by tumour associated monocytes correlated strongly with patient outcome independent of circulating tumour cell counts and the use of checkpoint inhibitors. We suggest that Pan cytokeratin staining within monocytes is an important indicator of tumour-associated inflammation post-therapy and an effective biomarker with strong prognostic capability for patient outcome.

**Supplementary Information:**

The online version contains supplementary material available at 10.1007/s10585-024-10270-w.

## Introduction

Non-small cell lung cancer (NSCLC) is the leading cause of cancer related mortality globally [1]. With increasing numbers of targeted and systemic therapies being developed, there is a need for the development of predictive biomarkers to track the efficacy of therapy in a real-time. Liquid biopsies, including blood sampling have been an important non-invasive means for evaluating aetiology and treatment response [2]. Moreover, comparing blood samples prior to and post therapy provides real-time longitudinal assessment of the tumour dynamics during the course of treatment. Mutational profiling of circulating tumour DNA (ctDNA) is a routine method to detect existing actionable mutations (e.g. *ALK, EGFR. KRAS, ERBB2*) as well as to track the emergence of new variants in NSCLC [3–5]. The availability of liquid biopsy-based diagnostics can be particularly appreciated in the metastatic setting where conventional tumour tissue sampling may be problematic due to difficult access and/or small size of lesions. Circulating tumour cells (CTCs) are the metastatic seed which travel through the blood and lympho-vasculature and lead to distant metastasis. With evolving CTC enrichment technologies, the capture efficiencies of CTCs has increased over the last few years with a number of microfluidic chip technologies [6–8]. At present, the CellSearch® system (Menarini Silicon Biosystems, Italy) is the only platform that has been approved by the FDA for the isolation of circulating tumor cells (CTCs). This method is mainly used to count CTCs of epithelial origin by positively selecting EpCAM + cells [9]. Over the past few years several studies reported that the transition from epithelial to mesenchymal (EMT) tumor cells mitigates the expression of epithelial markers like EpCAm and E-cadherin and increases the mesenchymal markers such as N-cadherin leading to label-dependent technologies fail to detect circulating tumor cells effectively [10, 11]. To overcome this obstacle, label-free technologies are gaining traction as possible platforms for investigating the size and deformability, two physical characteristics that distinguish tumor cells from WBCs in circulation, among others [12].

One alternative device that can sort cells by size utilizing a combination of inertial lift force and Dean drag forces is the spiral microfluidic device, which stands out among these technologies [13]. Running the samples through this device causes the cells occupy different lateral positions away from the walls of the microchannel, allowing for their separation at the bifurcation position [14]. Accordingly, cells larger than 14 μm like circulating tumor cells and their clusters flow into the “CTC outlet,” while cells smaller than this size flow into the “waste outlet” [15, 16].

Isolating CTCs from a variety of solid epithelial cancers, such as melanoma [17], head and neck [15], lung [18], and breast cancers [19] has been made possible by this method, which allows for the quick processing of large volumes of blood for CTC enrichment. CTC detection is a strong indicator of aggressive disease and therapy evasion resulting in poorer outcome [20].

Cancer progression and evolution facilitates changes in local and distant environments modulated by immune signalling. The crosstalk between tumour cells and the constituents of the immune system is incredibly complex as it combines tumour-suppressive as well as tumour-promoting mechanisms, most of which are still to be fully deciphered [21]. Macrophage polarisation—a biological process that gives rise to tumour-associated macrophages (TAMs)—has been long known to contribute to tumour-promoting microenvironment (TME) and metastasis [22]. In contrast, natural killer (NK) cells are traditionally considered to elicit a strong anti-cancer response [23]. More recently, however, this notion has been challenged by the overwhelming evidence of NK cell reprogramming by tumour cells and TME [23]. More notably, during cancer dissemination, CTCs can form clusters with platelets and/or neutrophils to escape NK surveillance [24–26]. Genome instability and the resulting high mutational load can cause tumour cells to generate immunogenic peptides—neoantigens [27, 28]. While neoantigens can stimulate CTC-neutralizing activity [29], cancer cells evolve to circumvent immune surveillance, reprogram and recruit immune cells to promote dissemination and metastatic seeding [30]. PD-L1-dependent evasion of immune surveillance is the most prominent example of such reprogramming. Checkpoint inhibition is one of the few successful attempts at harnessing the immune system for cancer therapy, however despite its successful use in NSCLC therapy, resistance mechanisms as well as the potential for immune-related adverse effects pose a great challenge and highlight the need for alternative strategies to assess therapy response beyond detection of PD-L1 expression (27, 31).

In this cross-sectional single-site study, we collected liquid biopsies from patients diagnosed with early, locally advanced, or metastatic lung cancer, undergoing surgery, or systemic therapy (chemotherapy/checkpoint inhibitors). Given the dynamic nature of the immune response, we aimed to investigate potential links between patient outcome, CTCs and their interactions with immune cells after administering therapy. While many CTC isolation protocols utilise positive enrichment strategies using EPCAM, we performed CTC enrichment with a microfluidics chip based on size exclusion.

## Methods

### Ethics approvals

Ethics approval was obtained from the Metro South Health District Human Research Ethics Committee in accordance with the National Health and Medical Research Council guidelines (HREC/11/QPAH/331) to collect samples from patients with resectable adenocarcinoma with either surgery or the combination of surgery and systemic therapy with agreement for a blood sample and follow-up at the Princess Alexandra Hospital. This study has institutional approval from the Queensland University of Technology human research ethics committee (1,100,001,420). Following written informed consent, 20 ml of blood was collected from n = 10 NSCLC patients undergoing systemic therapy (Table 1). Blood samples were enriched and characterised for circulating tumour cells (CTCs).

**Table 1:** Clinicopathological characteristics of patients’ samples for CTC populations

	Metrics	%
Age	38–79	
Gender		
Male	5	50
Female	5	50
Histopathology		
Adenocarcinoma	8	80
Squamous	1	10
Pleomorphic	1	10
Stage		
0	0	0
I	3	30
II	5	50
III	1	10
IV	1	10

### Spiral chip optimization

We examined the spiral chip with cancer cell lines to optimize its performance for CTC enrichment and recovery. Lung cancer cell lines A549 cell lines with three distinct cell counts (100, 500, and 1000 cells) were spiked into 5 ml of healthy blood, allowing the device to be optimized for CTC separation. Using the same processing procedure, spiked healthy controls were initially depleted of red blood cells and then diluted in PBS. To find the most appropriate flow rate for recovering the greatest number of cancer cells into the target outlet, cancer cell enrichment was done at five different flow rates of 1.3, 1.5, 1.7, 1.9, and 2.1. by comparing the quantity of recovered cancer cells and WBCs from different outlets We successfully separated spiking A549 cells from blood components with a high degree of recovery (85%) and lowest background cells at a flow rate of 1.7 ml/min.

### Setup and running of the spiral chip

**Fig. 1:**
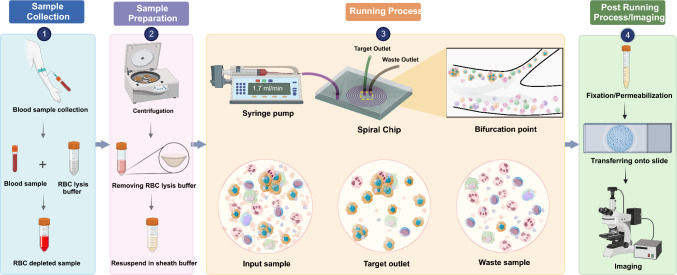
Workflow for CTC isolation. Enrichment of circulating tumor cells from lysed blood samples using a spiral chip. CTCs are separated from other blood cells regarding size differences and deformability

5 ml pf patient blood were collected in purple cap EDTA tubes, processed within 2 h and first depleted of red blood cells (RBC) by incubating with RBC lysis buffer (Astral Scientific, Taren Point, Australia). Cells were then centrifuged at 300 × g for 10 min and the pellet resuspended in 10 ml of phosphate buffered saline (PBS). Tygon® tubing (Saint-Gobain Corporation, Courbevoie, France) was inserted into the inlet/outlets of the spiral chip (Warkiani Lab, UTS, Sydney, Australia), and the inlet tubing connected to a syringe pump. The outlet tubing was connected to two sterile 15 ml collection tubes. An initial priming run was performed using PBS at a flow rate of 1.7 ml/min for 5 min. For CTC isolation, the RBC-depleted patient blood sample was loaded carefully into a 10 ml syringe (Terumo) and pumped through the spiral chip using the syringe pump at a flow rate of 1.7 ml/min. The outputs were collected, spun down at 300 × g for 5 min, and fixed with 4% paraformaldehyde solution. Finally, fixed cells were transferred onto histological slides using Cytospin 4 centrifuge (Thermo Fisher Scientific, Waltham, USA). Samples were then stored at 4 °C for subsequent immunofluorescent staining. Fig. 1.

### CTC immunostaining and characterisation

Cytospun cells were incubated overnight with Alexa Fluor®-conjugated primary antibodies (BioLegend®, San Diego, USA) against human cytokeratin 4, 5, 6, 8, 10, 13, 18 (pan-cytokeratin), CD45, and CD66b cell surface proteins. Antibodies were diluted in 5% w/v bovine serum albumin (Sigma-Aldrich, St. Louis, USA) in H2O to 1 µg/ml final concentration. Samples were washed with PBS (Thermo Fisher Scientific, Waltham, USA), followed by DNA staining with Hoechst 33,342 (Thermo Fisher Scientific, Waltham, USA). Fluorescent imaging was performed using IN Cell Analyzer 2200/6500HS (Cytiva, Marlborough, USA) at 20 × magnification covering the entire sample area. Image J software was used to analyse images and identify CTCs. A cell was deemed a CTC if it stained positive for pan-cytokeratin, Hoechst 33,342 and negative for CD45, CD66b.

## Results

### Patient characteristics

The clinicopathologic characteristics of the 10 patients with lung cancer are shown on Table 1. Age ranged from 38 to 76 with an average of 66.2 and median of 70.2 with an equal proportion of men and women. Histopathology revealed a majority of the patients’ cancers were adenocarcinoma (8/10) with half were stage 2 (5/10) and the remaining a mix of stage 1 and one each of late stage 3 and 4 (3/10).

### Isolation and detection of serum CTC biomarkers

**Fig. 2:**
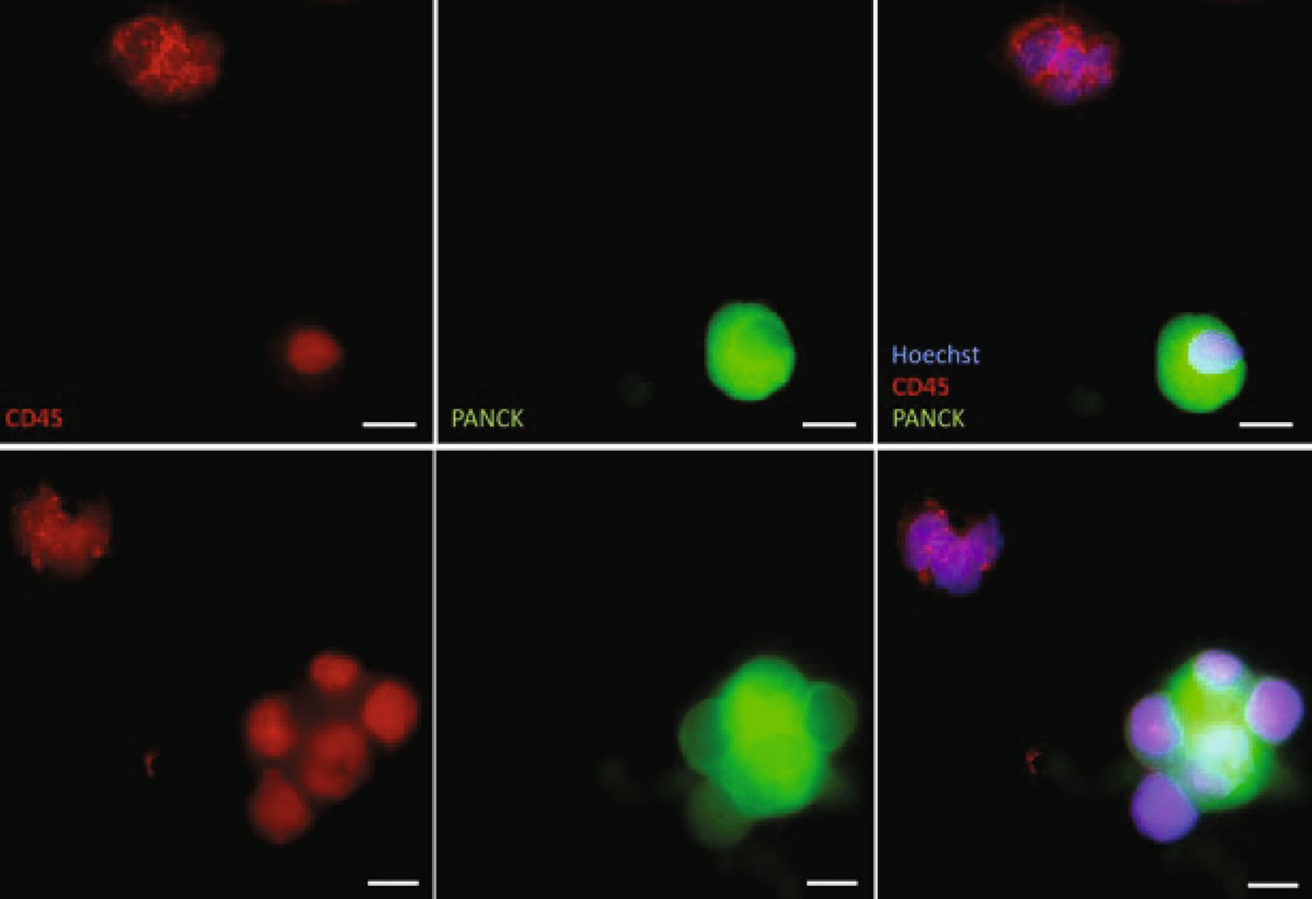
Staining of CTC populations. Representation of staining individual CTC populations (above) and CTC clusters (below). Color matched probed markers are labelled in the bottom left corner. Scale bar represents 5 uM

Peripheral blood mononuclear cells (PBMCs) were isolated and distinct cell populations identified utilizing spiral size exclusion and CD45, CD66b and Pan cytokeratin (PanCK) staining. We identified circulating cells that stain CD45 + on the cell surface (Fig. 2- upper left panel). Epithelial lung tumour cells (CTCs) PanCK + also displayed CD45 + nuclear staining (Fig. 2- upper centre panel), which was confirmed with DAPI staining (Fig. 2- upper right panel). PanCK + CTCs can also be observed in clusters (Fig. 2- lower panels).

### Cancer associated macrophage-like cells (CAML)

**Fig. 3:**
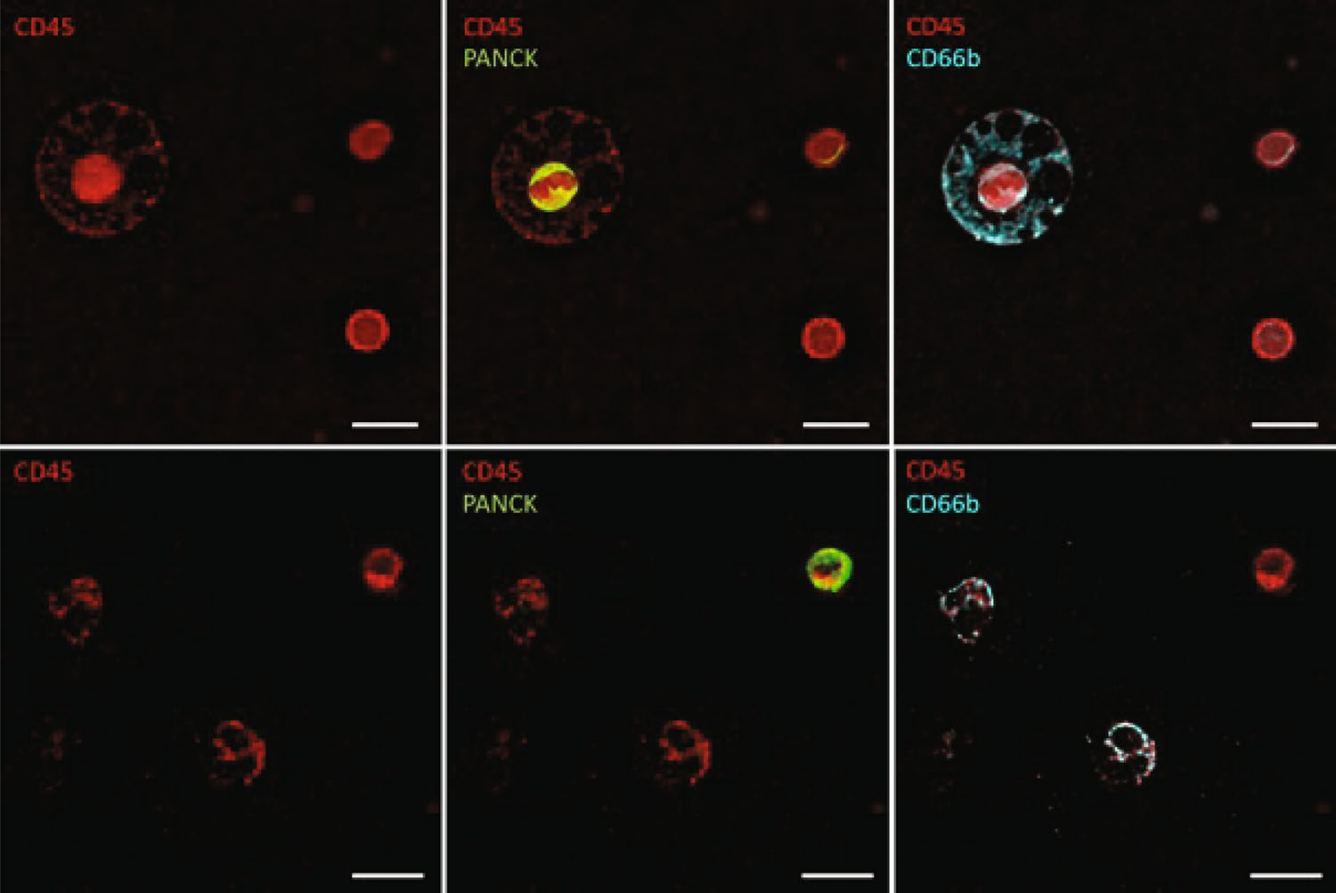
Cancer associated macrophage-like cells (CAMLs). Intracellular PanCK + suggesting engulfment of lung epithelial CTC can be associated with CD45 + /CD66b + neutrophil (top panels) or within CD45 + /CD66b- cancer associated macrophage-like cells (CAMLs)(lower panels). Scale bar represents 5 um

CTC are observed engulfed by phagocytotic cells in circulation [32]. We observed PanCK + staining within a CD45 + /CD66b + double positive activated neutrophil (Fig. 3- top panels). We also observed PanCK + staining within a CD45 + /CD66b-, likely cancer associated macrophage-like cells (CAML)[33](Fig. 3- lower panels).

### Confirmation of neutrophil association with CTCs

**Fig. 4:**
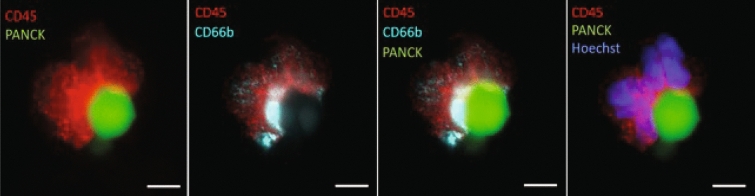
CTC parameters to determine patient outcome. Representation of a CD45 + /CD66b + nucleated cell tumour associated neutrophil (TAN) with positive cytokeratin staining for CTC. Color matched probed markers are labelled in the top left corner of each panel. Scale bar represents 5 um

A common observation in poor responding patients is the presence of circulating clusters of CTCs associated with neutrophils [25]. To confirm that PanCK + cells could be observed with tumour associated neutrophils we stained isolated PBMCs for CD66b and searched for an example associated with PanCK + . We observed a CD45 + /CD66b + cluster engulfing a PanCK + CTC (Fig. 4- left panel). The PanCK + cell is associated with a tumour associated neutrophil CD66b + (Fig. 4- next panel). We confirmed the neutrophil cluster to be nucleated with DAPI staining (Fig. 4- right panel).

### Stratification of patient groups with liquid biopsy parameters

**Fig. 5:**
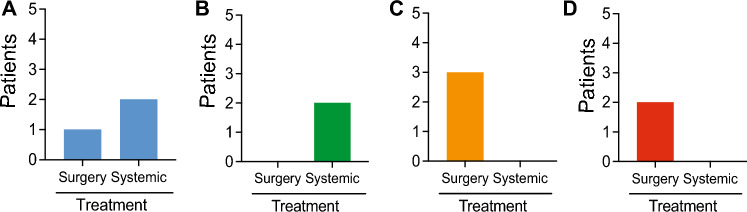
Treatment profile of stratified patients. Patients were stratified based upon CTC counts, clusters and staining. The resulting four independent groups treatment profiles are represented **A** mixed population of surgery alone and systemic therapy, **B** Systemic therapy only, **C** and **D** Surgery only

To correlate outcome of patients with liquid biopsy markers associated with immune and CTC interactions we evaluated the 10 patients across several indicators including, single CTC count, CTC clusters, CD45/66b + clusters, PanCK + inside CD45 + , presence CD66b + , CTCs/ml, clusters/ml, surgery versus systemic therapy and overall survival (months). Based on these indicators the 10 patients clustered into four groups (Supplementary Table 1). These four groups displayed a mixed population of surgery alone and systemic therapy (Fig. 5A), systemic therapy only (Fig. 5B), and surgery only (Fig. 5C/D).

**Fig. 6:**
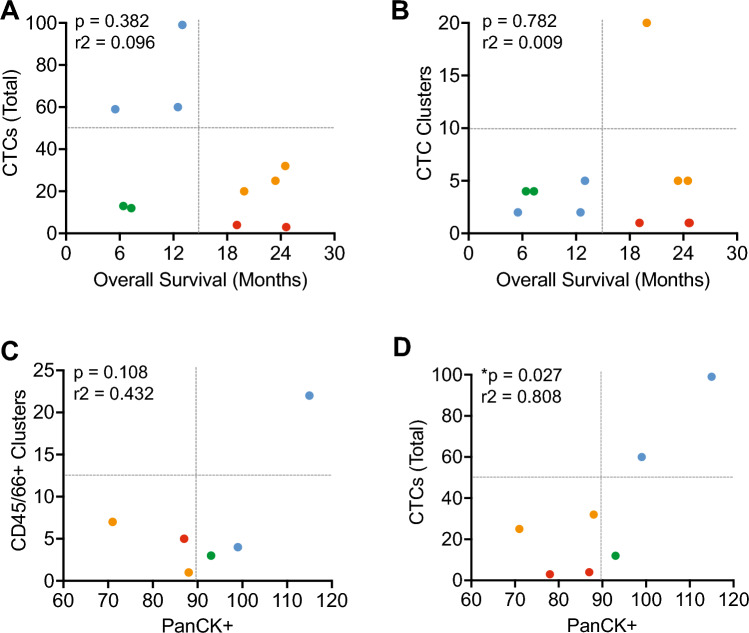
Clustering profile to segregate patient groups based on CTC parameters. **A** Clustered analysis comparing CTC count and overall survival. **B** Clustered analysis comparing CTC cluster count and overall survival. **C** Clustered analysis comparing cell clusters that stain double positive for CD45 and CD66b against CD45 positive cells that harbour intracellular PanCK staining. **D** Clustered analysis comparing CTC count and double positive for CD45 and CD66 against CD45 positive cells that harbour intracellular PanCK staining. The colors utilized in the plots represent the treatment profile of patients; blue- mixed population of surgery alone and systemic therapy, green- Systemic therapy only, yellow/red- Surgery only. Student ttest, two tailed p value stated. Pearsons correlation coefficient R squared value stated

To effectively stratify patient outcome with liquid biopsy parameters we hypothesized a relationship between poor patient survival and CTC counts. The patients treated with surgery alone, red labelled cohort displayed different CTC counts with low CTC counts and the yellow cohort for surgery, moderate CTC counts. Each of these groups displayed good patient survival metrics (Fig. 6A). The mixed group of surgery and systemic therapy (blue cohort) displayed the expected correlation between high CTC count and poor outcome (Fig. 6A). However, the final systemic therapy (green cohort) also with poor outcome displayed unexpectedly low CTC counts (Fig. 6A).

CTC clusters have been associated with poor patient outcome [24]. Overall CTC clusters correlated with outcome, except for a single patient with high cluster count and good outcome (Fig. 6B). This contradicts current clinical expectations [34]. We next evaluated immune antigen staining, comparing CD45/CD66b + double positive tumour associated neutrophil cluster counts, to tumour associated monocytes (TAMono) phagocytosis of lung cancer (PanCK + in CD45 +) (Fig. 6C—please note one of the red and one of the green cohorts each was not processed for this staining). Active PanCK + TAMono cells clustered the treatment groups, however CD45/66b + TAN cluster count was inconsistent across the blue cohort of patients, possibly caused by the difference in surgery versus systemic treatment of these two patients (Fig. 6C). Observing clustering of Active PanCK + TAMono cells, we compared this with overall CTC counts (Fig. 6D). This comparison improved the clustering of the mixed treatment (blue cohort), but not the exclusively systemically treated patients (green cohort) (Fig. 6D).

### Stratification of patient outcome

**Fig. 7:**
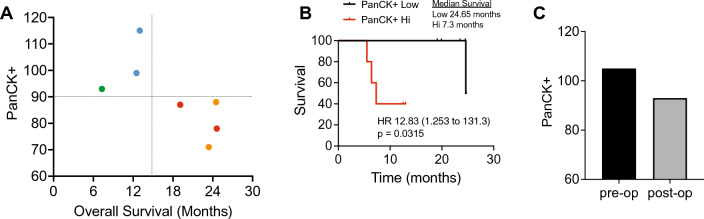
CTC parameters to determine patient outcome. **A** Clustered analysis comparing cells that harbour intracellular PanCK staining and overall survival. **B** Kaplan Meyer survival analysis comparing patients with low and high numbers of PanCK in CD45 + cells. **C** A comparison of PanCK in CD45 + cell count pre- and post-systemic therapy in a patient with poor outcome

Observing that overall CTC clusters and count did not correlate with outcome and PanCK + TAMono phagocytosis of lung cancer successfully separated the treatment cohorts (Fig. 7A). These metrics successfully demarcated the treatment populations. We then examined the stratification of patient survival rates utilizing active PanCK + TAMono, with a count of 90 for median cutoff (Fig. 7B). There was a significant difference between median survival with low patients surviving 24.65 months versus high surviving only 7.3 months. Hazard ratio exhibited 12 times risk of death for patients displaying high counts 90 + cells of PanCK + TAMono. Our processed liquid biopsy samples are post-surgery and post systemic therapy and it is speculated in the field that systemic therapies can enhance the numbers of PanCK + TAMono cells in circulation [35]. We evaluated the Active PanCK + TAMono cell count for a patient with poor outcome and observed a decrease after systemic chemotherapy (Fig. 7C).

## Discussion

Effective management of therapy requires close monitoring of treatment response so that protocols can be adjusted efficiently. For lung cancer (and other high-risk cancers), surveillance is achieved through periodic re-staging, using information from low-dose computed tomography scans [36]. This approach is sensitive, but not particularly specific, and unfortunately is utilized during the first 2 years following treatment where the greatest chance of recurrence occurs after curative intent [37]. As observed in our patient cohorts, curative intent can be divided into two distinct groups, surgery, and systemic therapy. Patients that underwent surgery alone presented with early-stage cancers (≤ stage 2), while those that received additional systemic therapy presented with later stage cancers (≥ stage 2). The clinical decisions for stage 2 were based upon nodal and metastatic involvement. Re-staging utilizing predictive biomarkers in liquid biopsies are showing promise for clinical utility, however low detection rates and prognostic value are two challenges faced when treating NSCLC that we have tackled in this study.

To evaluate CTC origin, the staining of surface antigen markers is routinely utilized. Antigen based capture of PBMC leukocytes is performed with CD45 + , while CTCs are defined with pan-cytokeratins (CK8/CK18/CK19) representing epithelial cells (CK^+^ and CD45^−^) [38]. DAPI^+^ can be utilized to mark and confirm nucleated cells [34]. Antigen-based isolation methods have been described to have poor sensitivity and specificity due to the CTC tendency for epithelial-to-mesenchymal transition (EMT), with a subsequent loss of epithelial surface markers such as EPCAM [39, 40]. The common use of EPCAM positivity to isolate CTCs was avoided in our study with isolation based upon the physical characteristics of the CTCs. While this widely used isolation and identification method is considered the gold standard for CTC detection, it has so far, only been approved by the FDA for routine use in metastatic breast, prostate, and colorectal cancer, but not yet in lung cancer [41]. Therefore, lung cancer still requires suitable prognostic markers.

Our results identified a prognostic value independent of CTC count, which is in contrast to several studies [6, 42–44]. PanCK^+^ CTC counts have been suggested to be a good marker to initially distinguish cancer patients for CTC enumeration, as counts are significantly higher in cancer patients than in healthy individuals [45]. Our results found a high ratio of PanCK + TAMono that is likely to consist of cancer associated macrophage-like cells CAMLs. These PanCK + cells detected per CTC accurately stratified outcome, alleviating the problems encountered by low detection rate. Circulating CAMLs are giant, fused hybrids of multi-nucleated stromal macrophages (of myeloid origin; CD14 + /CD11c +) with a sizeable atypical nucleus and positivity for the epithelial markers with cytokeratin-positive cytoplasm found in the peripheral blood of patients with solid tumours [32, 46]. These cells are reported to be associated with the development of metastasis by acquiring features such as genetic/epigenetic heterogeneity, immune tolerance, and chemotherapeutic resistance [32].

The presence of tumour associated neutrophils (TAN CD66b +) associated with CD45 + and PanCK + CTCs in our samples suggests active suppression of immune surveillance, and the antitumor response of effector T cells [47–49]. CTCs that are significantly associated with neutrophils in both mouse models and breast cancer patients, exhibit more metastatic potential with greater expression of genes that involve cell cycle progression compared to CTCs alone [25]. CTCs clusters with neutrophils anchor to the vascular endothelium for extravasation while resisting shear stress, with the process being mediated by a series of cell adhesion proteins, including cadherin, integrin, and surface glycoprotein [50, 51]. However, CD45/66b + TAN cluster count was inconsistent across patients displaying poor outcome and although a marker of immune modulation we and others were not able to correlate CD66b + with outcome [52].

The current held opinion in utilizing liquid biopsy for monitoring of treatment response, is that there usually a postsurgical increase in CTC count, however it has been observed for various cancer types that CTC counts normalize and often decrease after surgery [53], suggesting shedding from the primary tumour is a larger reservoir than surgical disruption. Similar observations have been observed with systemic therapies. Systemic therapy is delivered in the form of chemotherapy (platinum agents, antimetabolites, antineoplastic, topoisomerase inhibitors and antiangiogenic agents) and immunotherapy antibodies. Recent studies have shown that chemotherapy destabilizes the blood vasculature and can increase CTC influx into the circulation of metastatic cancer patients [54]. CTC level was found to increase two-fold from the initial pre-treatment level after 1 cycle of chemotherapy and returned to baseline after 2 cycles of chemotherapy, most notably utilizing platinum agents and antimetabolites [54]. We observed a reduction in CTC count post-treatment in our matched systemic therapy treated patient. Therefore, we suggest that liquid biopsies soon after initial surgery or systemic therapy may represent elevated false negative outcome counts that are not truly indicative of response and should be delayed or repeated to provide more informative results. In the scenario of immune modulating systemic therapy, the response is suggested to be directly correlated with PD-L1-positive CTC counts [55]. Gene expression analysis has revealed that the higher levels of PD-L1 are associated with poorer prognosis [56] and in breast cancer the CTC numbers were decreased, as a signature of the successful immunotherapy [57]. In addition, after discontinuing immunotherapy, the percentage of the PD-L1-positive CTC's continuously increased and a recurrence disease state was noted [57]. CTCs can therefore be used to monitor dynamic changes in response to PD-L1 during immunotherapy [58]. However, their use in our systemic patients was restricted to locally advanced and metastatic cases or as part of a clinical trial where the selection criteria are met [59]. Four patients whose blood samples were collected as part of this study, developed immune related adverse events, and had to cease immune checkpoint therapy. Therefore, there is potential for PD-L1 as a marker but we suggest that treatment tolerability is a limiting factor for standardized care.

Patients with CTC counts can generally be expected to have a poorer outcome however, we find that patients with low CTC counts do not necessarily have a good outcome. Our study supports the mechanism that immune response in the circulation is an important indicator of tumour-associated inflammation post-therapy and an effective detection method with strong prognostic capability for patient outcome.

### Supplementary Information

Below is the link to the electronic supplementary material.

Supplementary file1 (DOCX 582 KB)

## References

[1] Molina JR, Yang P, Cassivi SD, Schild SE, Adjei AA (2008). Non-small cell lung cancer: epidemiology, risk factors, treatment, and survivorship. Mayo Clin Proc.

[2] Lone SN, Nisar S, Masoodi T, Singh M, Rizwan A, Hashem S (2022). Liquid biopsy: a step closer to transform diagnosis, prognosis and future of cancer treatments. Mol Cancer.

[3] Cho BC, Loong HHF, Tsai CM, Teo MLP, Kim HR, Lim SM (2022). Genomic landscape of non-small cell lung cancer (NSCLC) in East Asia using circulating tumor DNA (ctDNA) in clinical practice. Curr Oncol.

[4] Mathios D, Johansen JS, Cristiano S, Medina JE, Phallen J, Larsen KR (2021). Detection and characterization of lung cancer using cell-free DNA fragmentomes. Nat Commun.

[5] Fernandes MGO, Cruz-Martins N, Machado JC, Costa JL, Hespanhol V (2021). The value of cell-free circulating tumour DNA profiling in advanced non-small cell lung cancer (NSCLC) management. Cancer Cell Int.

[6] Kapeleris J, Kulasinghe A, Warkiani ME, Vela I, Kenny L, O’Byrne K (2018). The prognostic role of circulating tumor cells (CTCs) in lung cancer. Front Oncol.

[7] Zhou J, Kulasinghe A, Bogseth A, O’Byrne K, Punyadeera C, Papautsky I (2019). Isolation of circulating tumor cells in non-small-cell-lung-cancer patients using a multi-flow microfluidic channel. Microsystems Nanoeng.

[8] Kulasinghe A, Lim Y, Kapeleris J, Warkiani M, O’Byrne K, Punyadeera C (2020). The use of three-dimensional DNA fluorescent in situ hybridization (3D DNA FISH) for the detection of anaplastic lymphoma kinase (ALK) in non-small cell lung cancer (NSCLC) circulating tumor cells. Cells.

[9] Riethdorf S, O’Flaherty L, Hille C, Pantel K (2018). Clinical applications of the cell search platform in cancer patients. Adv Drug Deliv Rev.

[10] Zhang Z, Wuethrich A, Wang J, Korbie D, Lin LL, Trau M (2021). Dynamic monitoring of EMT in CTCs as an indicator of cancer metastasis. Anal Chem.

[11] Kitz J, Goodale D, Postenka C, Lowes LE, Allan AL (2021). EMT-independent detection of circulating tumor cells in human blood samples and pre-clinical mouse models of metastasis. Clin Exp Metastasis.

[12] Yaghoubi Naei V, Bordhan P, Mirakhorli F, Khorrami M, Shrestha J, Nazari H (2023). Advances in novel strategies for isolation, characterization, and analysis of CTCs and ctDNA. Ther Adv Med Oncol.

[13] Warkiani ME, Khoo BL, Wu L, Tay AKP, Bhagat AAS, Han J (2016). Ultra-fast, label-free isolation of circulating tumor cells from blood using spiral microfluidics. Nat Protoc.

[14] Warkiani ME, Khoo BL, Tan DSW, Bhagat AAS, Lim WT, Yap YS (2014). An ultra-high-throughput spiral microfluidic biochip for the enrichment of circulating tumor cells. Analyst.

[15] Kulasinghe A, Tran THP, Blick T, O’Byrne K, Thompson EW, Warkiani ME (2017). Enrichment of circulating head and neck tumour cells using spiral microfluidic technology. Sci Rep.

[16] Kulasinghe A, Zhou J, Kenny L, Papautsky I, Punyadeera C (2019). Capture of circulating tumour cell clusters using straight microfluidic chips. Cancers (Basel).

[17] Aya-Bonilla CA, Marsavela G, Freeman JB, Lomma C, Frank MH, Khattak MA (2017). Isolation and detection of circulating tumour cells from metastatic melanoma patients using a slanted spiral microfluidic device. Oncotarget.

[18] Kulasinghe A, Kapeleris J, Cooper C, Warkiani ME, O’Byrne K, Punyadeera C (2019). Phenotypic characterization of circulating lung cancer cells for clinically actionable targets. Cancers (Basel).

[19] Warkiani ME, Guan G, Luan KB, Lee WC, Bhagat AAS, Kant Chaudhuri P (2014). Slanted spiral microfluidics for the ultra-fast, label-free isolation of circulating tumor cells. Lab Chip.

[20] Lawrence R, Watters M, Davies CR, Pantel K, Lu YJ (2023). Circulating tumour cells for early detection of clinically relevant cancer. Nat Rev Clin Oncol.

[21] Sutton TL, Patel RK, Anderson AN, Bowden SG, Whalen R, Giske NR (2022). Circulating cells with macrophage-like characteristics in cancer: the importance of circulating neoplastic-immune hybrid cells in cancer. Cancers (Basel).

[22] Lin Y, Xu J, Lan H (2019). Tumor-associated macrophages in tumor metastasis: biological roles and clinical therapeutic applications. J Hematol Oncol.

[23] Chan IS, Ewald AJ (2022). The changing role of natural killer cells in cancer metastasis. J Clin Invest.

[24] Ivanova E, Ward A, Wiegmans AP, Richard DJ (2020). Circulating tumor cells in metastatic breast cancer: from genome instability to metastasis. Front Mol Biosci.

[25] Szczerba BM, Castro-Giner F, Vetter M, Krol I, Gkountela S, Landin J (2019). Neutrophils escort circulating tumour cells to enable cell cycle progression. Nature.

[26] Herath S, Razavi Bazaz S, Monkman J, Ebrahimi Warkiani M, Richard D, O’Byrne K (2020). Circulating tumor cell clusters: insights into tumour dissemination and metastasis. Expert Rev Mol Diagn.

[27] Riaz N, Morris L, Havel JJ, Makarov V, Desrichard A, Chan TA (2016). The role of neoantigens in response to immune checkpoint blockade. Int Immunol.

[28] Germano G, Lamba S, Rospo G, Barault L, Magri A, Maione F (2017). Inactivation of DNA repair triggers neoantigen generation and impairs tumour growth. Nature.

[29] Liu WR, Fisher DE (2021). Epitope spreading and the efficacy of immune checkpoint inhibition in cancer. Int J Oncol Res.

[30] Leone K, Poggiana C, Zamarchi R (2018). The interplay between circulating tumor cells and the immune system: from immune escape to cancer immunotherapy. Diagnostics.

[31] Shiravand Y, Khodadadi F, Kashani SMA, Hosseini-Fard SR, Hosseini S, Sadeghirad H (2022). Immune checkpoint inhibitors in cancer therapy. Curr Oncol.

[32] Adams DL, Martin SS, Alpaugh RK, Charpentier M, Tsai S, Bergan RC (2014). Circulating giant macrophages as a potential biomarker of solid tumors. Proc Natl Acad Sci USA.

[33] Sulaiman R, De P, Aske JC, Lin X, Dale A, Vaselaar E (2022). Identification and morphological characterization of features of circulating cancer-associated macrophage-like cells (CAMLs) in endometrial cancers. Cancers (Basel).

[34] Maly V, Maly O, Kolostova K, Bobek V (2019). Circulating tumor cells in diagnosis and treatment of lung cancer. In Vivo.

[35] Cassetta L, Fragkogianni S, Sims AH, Swierczak A, Forrester LM, Zhang H (2019). Human tumor-associated macrophage and monocyte transcriptional landscapes reveal cancer-specific reprogramming, biomarkers, and therapeutic targets. Cancer Cell.

[36] Sun Z, Li P, Wu Z, Li B, Li W, Zhao M (2022). Circulating CD45+EpCAM+ cells as a diagnostic marker for early-stage primary lung cancer. Front Med Technol.

[37] Westeel V (2018). MS17.03 surveillance and second primary malignancies in lung cancer survivors. J Thorac Oncol.

[38] Cristofanilli M, Budd GT, Ellis MJ, Stopeck A, Matera J, Miller MC (2004). Circulating tumor cells, disease progression, and survival in metastatic breast cancer. N Engl J Med.

[39] Duda DG, Duyverman AMMJ, Kohno M, Snuderl M, Steller EJA, Fukumura D (2010). Malignant cells facilitate lung metastasis by bringing their own soil. Proc Natl Acad Sci USA.

[40] Joosse SA, Gorges TM, Pantel K (2015). Biology, detection, and clinical implications of circulating tumor cells. EMBO Mol Med.

[41] Kemper M, Krekeler C, Menck K, Lenz G, Evers G, Schulze AB (2023). Liquid biopsies in lung cancer. Cancers.

[42] Li Z, Xu K, Tartarone A, Santarpia M, Zhu Y, Jiang G (2021). Circulating tumor cells can predict the prognosis of patients with non-small cell lung cancer after resection: a retrospective study. Transl Lung Cancer Res.

[43] Wang PP, Liu SH, Te CC, Lv L, Li D, Liu QY (2020). Circulating tumor cells as a new predictive and prognostic factor in patients with small cell lung cancer. J Cancer.

[44] Krebs MG, Sloane R, Priest L, Lancashire L, Hou JM, Greystoke A (2011). Evaluation and prognostic significance of circulating tumor cells in patients with non-small-cell lung cancer. J Clin Oncol.

[45] Lu SH, Tsai WS, Chang YH, Chou TY, Pang ST, Lin PH (2016). Identifying cancer origin using circulating tumor cells. Cancer Biol Ther.

[46] Manjunath Y, Porciani D, Mitchem JB, Suvilesh KN, Avella DM, Kimchi ET (2020). Tumor-cell–macrophage fusion cells as liquid biomarkers and tumor enhancers in cancer. Int J Mol Sci.

[47] Kumagai Y, Ohzawa H, Miyato H, Horie H, Hosoya Y, Lefor AK (2020). Surgical stress increases circulating low-density neutrophils, which may promote on tumor recurrence. J Surg Res.

[48] Zhang J, Qiao X, Shi H, Han X, Liu W, Tian X (2016). Circulating tumor-associated neutrophils (cTAN) contribute to circulating tumor cell survival by suppressing peripheral leukocyte activation. Tumor Biol.

[49] Spiegel A, Brooks MW, Houshyar S, Reinhardt F, Ardolino M, Fessler E (2016). Neutrophils suppress intraluminal NK cell-mediated tumor cell clearance and enhance extravasation of disseminated carcinoma cells. Cancer Discov.

[50] Huh SJ, Liang S, Sharma A, Dong C, Robertson GP (2010). Transiently entrapped circulating tumor cells interact with neutrophils to facilitate lung metastasis development. Cancer Res.

[51] Spicer JD, McDonald B, Cools-Lartigue JJ, Chow SC, Giannias B, Kubes P (2012). Neutrophils promote liver metastasis via Mac-1-mediated interactions with circulating tumor cells. Cancer Res.

[52] Carus A, Ladekarl M, Hager H, Pilegaard H, Nielsen PS, Donskov F (2013). Tumor-associated neutrophils and macrophages in non-small cell lung cancer: No immediate impact on patient outcome. Lung Cancer.

[53] Kaifi JT, Li G, Clawson G, Kimchi ET, Staveley-O’Carroll KF (2016). Perioperative circulating tumor cell detection: current perspectives. Cancer Biol Ther.

[54] Ortiz-Otero N, Marshall JR, Lash B, King MR (2020). Chemotherapy-induced release of circulating-tumor cells into the bloodstream in collective migration units with cancer-associated fibroblasts in metastatic cancer patients. BMC Cancer.

[55] Rzhevskiy A, Kapitannikova A, Malinina P, Volovetsky A, Es HA, Kulasinghe A (2021). Emerging role of circulating tumor cells in immunotherapy. Theranostics.

[56] Wang Y, Kim TH, Fouladdel S, Zhang Z, Soni P, Qin A (2019). PD-L1 Expression in circulating tumor cells increases during radio(chemo)therapy and indicates poor prognosis in non-small cell lung cancer. Sci Rep.

[57] Schott DS, Pizon M, Pachmann U, Pachmann K (2017). Sensitive detection of PD-L1 expression on circulating epithelial tumor cells (CETCs) could be a potential biomarker to select patients for treatment with PD-1/PD-L1 inhibitors in early and metastatic solid tumors. Oncotarget.

[58] Nicolazzo C, Raimondi C, Mancini M, Caponnetto S, Gradilone A, Gandini O (2016). Monitoring PD-L1 positive circulating tumor cells in non-small cell lung cancer patients treated with the PD-1 inhibitor Nivolumab. Sci Rep.

[59] Pharmaceutical Benefits Scheme (PBS) 8107635

