# Supplementary material for: Poor patient outcome correlates with active engulfment of cytokeratin positive CTCs within cancer-associated monocyte population in lung cancer
Source: Clin Exp Metastasis. 2024 Feb 28;41(3):219–28. doi: 10.1007/s10585-024-10270-w (PMC11213738; doi:10.1007/s10585-024-10270-w)
Supplement: Supplementary file 1 — Supplementary file1 (DOCX 582 KB) [file 10585_2024_10270_MOESM1_ESM.docx]

***Supplementary Table 1. Patient Immuno-clinical metrics.***


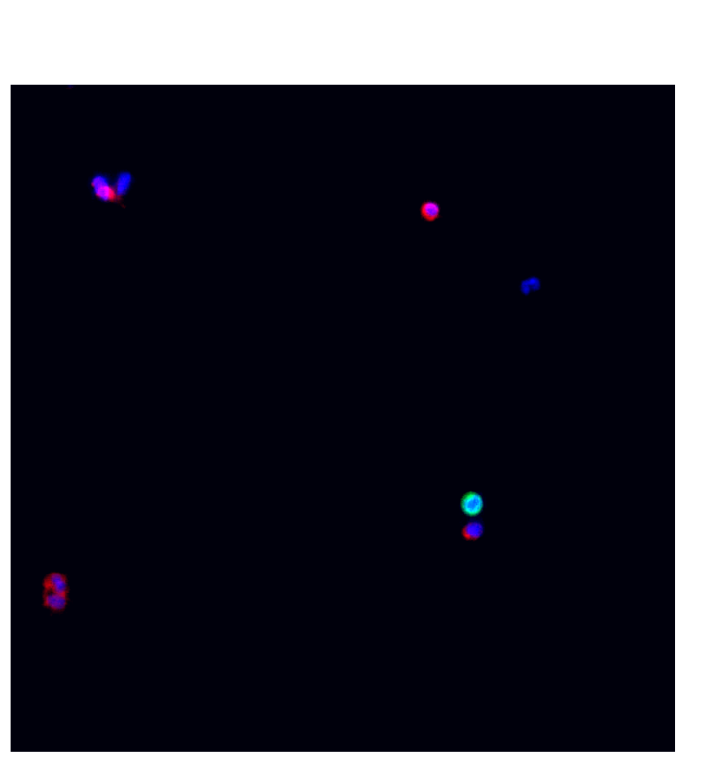


B


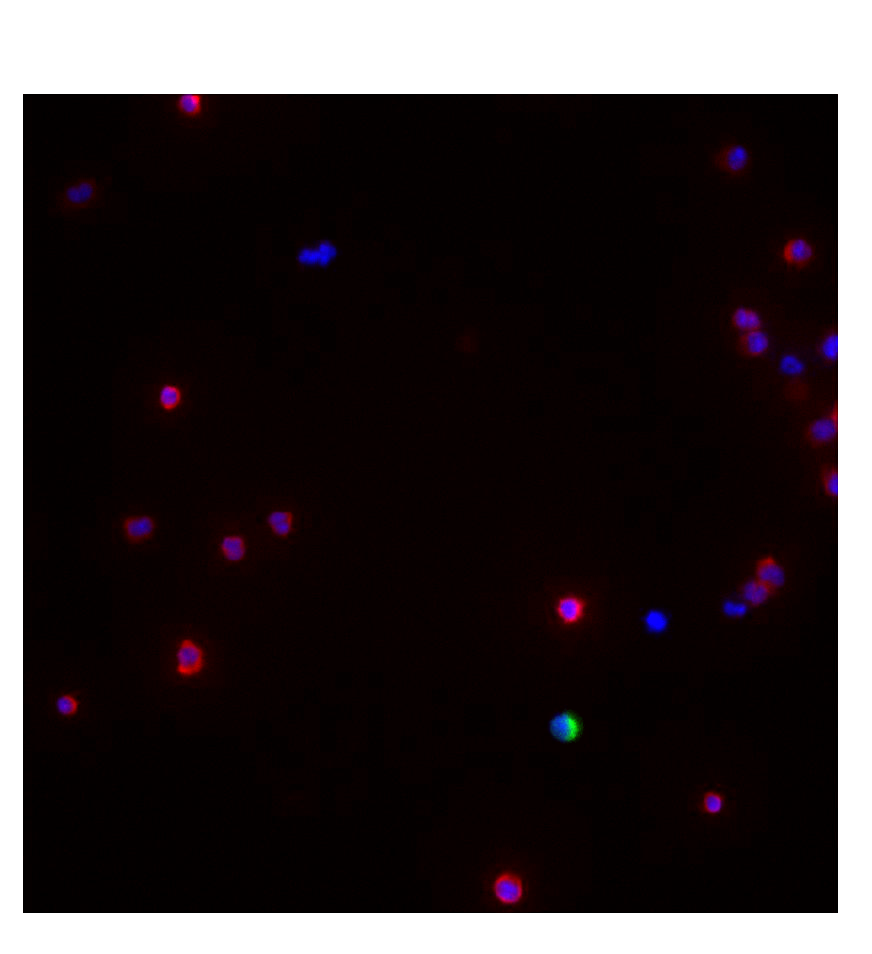


A

DAPI

PanCK

CD45

***Figure S1. Optimization of enrichment procedure.*** ***A****) High background cell in the target outlet before optimization.* ***B****) Reduced background cells in the target outlet at the optimal flow rate. Yellow arrows = white blood cells, White arrows = Cancer cells*
